# Cope PPA—Adaptation of the Biographic-Narrative Approach for Persons with Primary Progressive Aphasia: Protocol for Clinical Trial Design

**DOI:** 10.3390/brainsci14070692

**Published:** 2024-07-11

**Authors:** Mirjam Gauch, Sabine Corsten, Anna-Lena Köb, Oliver Tüscher, Isabel Heinrich, Katharina Geschke

**Affiliations:** 1 Department of Psychiatry and Psychotherapy, University Medical Center of the Johannes Gutenberg University Mainz, 55131 Mainz, Germany; anna-lena.koeb@unimedizin-mainz.de (A.-L.K.); tuescher@uni-mainz.de (O.T.); iheinric@uni-mainz.de (I.H.); kgeschke@uni-mainz.de (K.G.); 2 Faculty of Healthcare and Nursing, Catholic University of Applied Sciences Mainz, 55122 Mainz, Germany; sabine.corsten@kh-mz.de

**Keywords:** primary progressive aphasia, biographic-narrative approach, reminiscence, life review, life-review therapy, quality of life

## Abstract

Persons with primary progressive aphasia (PPA) often experience limitations in their quality of life (QoL). Some studies have shown positive effects of speech and language therapy on QoL in persons with PPA. However, there is still a lack of evidence for disorder-specific approaches for this important therapeutic goal. The biographic-narrative approach (narraktiv) has been shown to significantly improve QoL in persons with post-stroke aphasia. In the planned study, the biographic-narrative approach will be adapted for persons with PPA (Cope PPA), and its efficacy will be investigated. First, a focus group interview with five persons with PPA will be conducted to identify the wishes and needs of participants. Based on the results, the narraktiv manual according to Corsten et al. (2015) will be revised. Second, an efficacy study will be conducted according to the new Cope PPA manual with 24 persons with PPA in a waiting group control design. The primary outcome, QoL, will be assessed using questionnaires (Stroke and Aphasia Quality of Life Scale-39) and semistructured interviews. Depressive symptoms, life satisfaction and cognitive/communicative functioning will also be assessed. If Cope PPA proves efficacy, this study may help to improve the treatment of persons with PPA.

## 1. Introduction

Primary progressive aphasia (PPA) is a rare clinical syndrome characterised by prominent speech disorder and an average prevalence of 3:100,000 [1,2]. It is classified into three variants (nonfluent, semantic, and logopenic) and is associated with Alzheimer’s pathology or frontotemporal dementia [3,4]. As the age of onset is approximately 65 years [3], it has a devastating impact on the professional and private roles of an affected person [5]. The treatment of PPA is limited to mitigating symptoms, as no curative therapy is known to date [6]. About 40% of persons with PPA develop depression as a comorbidity during the course of the disease, which can be attributed to a perceived awareness of the individual situation and to an impaired self-image of a person with a speech disorder [7,8,9]. Since aphasia limits access to psychotherapeutic services [10], approaches that enhance quality of life (QoL) are increasingly being considered by speech and language therapists (SLTs). Further, QoL, which is defined by the World Health Organisation (WHO) as an individual’s perception of their position in life considering cultural and personal context factors in relation to personal goals and concerns [11], is described as the “ultimate” outcome of aphasia therapy [12]. Psychoeducational group programs and participatory approaches have already been successfully applied to persons with PPA and are valued by individuals [13,14]. Counselling and communication partner training showed positive effects on coping with illness and communication in dyads [15,16]. However, there is uncertainty in the application of QoL-related methods in persons with PPA and a lack of high-quality studies proving effects on QoL of this target group [7,17].

In a systematic review summarising rehabilitative interventions for the prevention and treatment of depression in poststroke aphasia patients, the authors refer to a mixed-methods study by Corsten et al. (2015) [18].

This study is emphasised as the one with the highest quality and level of evidence for persons with poststroke aphasia and demonstrates the effects of the biographic-narrative approach narraktiv [18,19]. The narraktiv intervention contains five individual and seven group therapy sessions in which biographic storytelling is stimulated. This approach is based on the idea that aphasia hinders the natural and daily redevelopment of identity, as the medium for identity work (communication) is impaired. Disturbed identity work leads to a negative self-image and thus reduces QoL. Supporting narration can help persons with aphasia to work on their identity and to build a sense of coherence, ultimately improving their QoL [9,20,21]. The participants in the narraktiv intervention were persons with poststroke aphasia who did not have depression or who exhibited early signs of depression. Significant improvements with small to medium effect sizes were observed in improvement of mood and health-related QoL, measured by the Visual Analogue Mood Scale (VAMS, [22]) and the Aachen Life Quality Inventory [23]. The effects remained stable over a period of three months after the intervention or even increased towards follow-up, as in the case of the Aachen Life Quality Inventory scores. Since many therapeutic approaches can be transferred from persons with poststroke aphasia to persons with PPA [6], it seems promising to apply the concept of the biographic-narrative approach narraktiv according to Corsten et al. (2015) to persons with PPA.

Comparable approaches such as ‘simple reminiscence’, ‘life-review’, or ‘life-review therapy’ [24,25] have been used successfully in patients with dementia and older persons [24,26,27,28]. While ‘simple reminiscence’ focuses on the recall and sharing of memories, ‘life review’ is more evaluative and aims to cover the whole life story chronologically. ‘Life-review therapy’ is a continuation of ‘life review’, in which more elements of cognitive therapy are used to give persons a more positive view of their lives [26]. Although ‘simple reminiscence’ can be conducted in a nursing home setting by counsellors with basic skills, ‘life review’ should be directed towards individuals with mild psychological distress and delivered by counsellors with advanced skills. In cases of serious psychological distress, experienced counsellors could use ‘life-review therapy’ [25]. Another way to differentiate between the approaches is to categorise them as structured or unstructured therapies [29]. The narraktiv intervention is most likely to be categorised between structured/unstructured approaches and combines aspects of ‘simple reminiscence’, ‘life review’, and ‘life-review therapy’. On the one hand, it is highly structured for the therapist through predetermined question types and a fixed structure of individual and group therapy sessions; on the other hand, it is nondirective. For example, participants are free to decide what they want to tell about their own lives and what personal relevance they want to have, thanks to the open-narrative stimulus. The biography does not have to be presented in every detail, neither chronologically nor in terms of content. The focus on persons with aphasia distinguishes the biographic-narrative approach from reminiscence or life-review therapy, which were developed for persons with memory deficits rather than for persons with primary aphasia [30].

Due to its person-centred focus on QoL, narraktiv differs from the participation- and environment-based group programmes that try to enhance QoL indirectly and have been evaluated for persons with PPA to date [13,31]. Because of the non-directive character, the approach also differs from counselling services that have been evaluated for use with persons with PPA and their caregivers [15]. As far as we are aware, there is only one case study indicating the use of narraktiv in a person with an advanced nonfluent variant of PPA in a palliative care setting [32]. The adjustments and effects of narraktiv are not described by the authors [32]. However, it seems close at hand that the original narraktiv intervention which is categorised by experts as a rehabilitative care intervention [18], needs to be adapted for persons with PPA who may have other needs against the background of cognitive deficits and the progression of the disease. Participatory research enables development according to a bottom-up principle [33]. In our study, it will be used to make the approach more sustainable and acceptable.

The aim of the present study is to prove the transferability of the biographic-narrative approach to the treatment of persons with PPA. In a focus-group interview, the potential need to adapt the biographic-narrative approach narraktiv according to Corsten et al. (2015) for persons with PPA will be discussed. An efficacy study will then be conducted to evaluate the efficacy of the adapted biographic-narrative approach Cope PPA for persons with PPA and to explore the effects in individual cases.

The research questions are as follows:

1. What need for adaptation do persons with PPA see for the biographic-narrative approach narraktiv for use with persons with PPA?
2. Does the adapted biographic-narrative approach Cope PPA improve the QoL of persons with PPA?

## 2. Materials and Methods

In the following, the method of the planned study is reported considering the SPIRIT checklist. This international checklist contains 33 evidence-based recommended items, reflecting the minimum content of a clinical trial protocol (2013, see Additional Supplementary Materials File S1). The current study is monocentric, as it will be conducted only at the outpatient memory clinic at the Department of Psychiatry and Psychotherapy of the University Hospital Mainz (Mainz, Germany). The study complies with the General Data Protection Regulations and will be conducted in accordance with the Declaration of Helsinki. This study was approved by the Ethics Committee of the Federal Medical Association of Rhineland-Palatinate (2023-17294).

### 2.1. Inclusion and Exclusion Criteria

Only persons who fulfil the clinical diagnostic consensus criteria for PPA according to Gorno-Tempini (2011, [4]) will be included in both phases of the study. Persons with all variants of PPA are eligible. Although persons with all stages of PPA can be included, participants must be willing to work in a group and be able to communicate basic thoughts. People with mutism were therefore excluded from both phases of the study. According to the consensus criteria [4], persons were excluded if their speech deficits could be better explained by other neurological disorders like stroke or brain injury. Further exclusion criteria were inadequate visual acuity despite correction with a visual aid and inadequate hearing despite the use of a hearing aid.

The ability to give consent is a prerequisite for participation in both phases of the study. For persons with dementia syndromes such as PPA, there may be uncertainties in clarifying the ability to consent [34]; for this reason, the following criteria of the guideline ‘capacity to consent of persons with dementia’ are used for this purpose [35]. The consent of the participants will be obtained in detail and in an understandable form by the deputy study director (M.G.) and study doctors involved (K.G., I.H.) before the start of the study, taking into account the linguistic, cognitive, and emotional competences of each participant. Immediately before each assessment and therapy session, the ability to give consent will be reassessed and documented by the research team based on the following criteria: (1) understanding relevant information, (2) understanding the implications for one’s own situation, (3) reasoning about the information, and (4) expressing a treatment choice [34,36]. Participants will give their informed consent to participate and to be video-recorded during the assessments and the therapy sessions. The participants’ faces will be fully visible to account for the participants’ facial expressions as a form of communication. For the efficacy study, caregivers will give their informed consent for being interviewed at the end of the intervention.

Persons with pronounced cognitive deficits, corresponding to a score < 10 out of 30 points in the established cognitive screening test Mini Mental-State Examination (MMSE, [37]), will be excluded at the beginning of the efficacy study, as there are indications from the literature that more complex questionnaires cannot be answered in cases of severe cognitive impairment [38]. Individuals whose cognition deteriorates to <10 points on the MMSE over the course of the study will not immediately be excluded. In these cases, an individual decision will be based on clinician ratings and according to the participants’ wishes.

Furthermore, the presence of severe depressive symptoms (clinical diagnosis and/or severe depressive symptoms, corresponding to >35 points on the Montgomery–Åsberg Depression Rating Scale (MADRS, [39]) is defined as an exclusion criterion at the beginning of the study because narraktiv was developed for preventing the occurrence of depression and has not been evaluated for the treatment of this condition [19]. However, due to the positive effects of participating in peer support groups on depression, persons with mild to moderate depression will not be excluded from the present study [40]. If participants show signs of depression in the course of the study (MADRS > 35 at t1–t3 or characteristics of a depressive disorder such as physical exhaustion, appetite disorders, changes in gestures/facial expressions or physiognomy during the therapy sessions), suicidal tendencies will be explored and documented in an individual setting. Questions from the national guideline “Unipolar depression” will be used for this purpose, e.g., “Have you had or do you have concrete plans to harm yourself?” and “Is there anything that keeps you alive?” [23]. If acute suicidal behaviour is suspected, an emergency psychiatric counselling will be conducted at the Department of Psychiatry and Psychotherapy of the University Hospital Mainz. If there is no acute suicidal tendency, the person will be promptly seen by a specialist in the outpatient memory clinic and the research team will plan further treatment steps if necessary. Participation in the study will be in addition to any other treatment participants may receive.

Whether persons with PPA previously received or currently receive speech and language therapy will not be a criterion for inclusion or exclusion in our study.

### 2.2. Identification and Recruitment of Participants

Participants will be recruited via the outpatient memory clinic as well as by cooperating practices and outpatient speech and language therapy practices. A recruitment flyer has been created in understandable, simple language to inform participants about the study. Prior to participation, potential participants will be screened through face-to-face counselling.

### 2.3. Design

**Figure 1:**
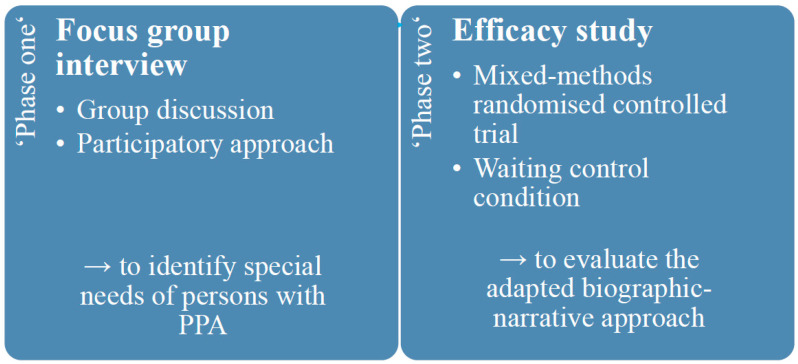
Study design including ‘phase one’ and ‘phase two’.

The planned study consists of two phases using an adaptive clinical trial design. ‘Phase one’ envisages a focus-group interview in which five persons with PPA will take part. The aim of the focus-group interview is to identify specific needs of persons with PPA for adaptation of the biographic-narrative approach narraktiv for use with persons with PPA. The ‘phase one’ corresponds to a qualitative exploratory study based on the principle of a group discussion, aiming to generate data based on social interaction involving persons with PPA in the study process in the sense of participatory research [41]. In ‘phase two’, an efficacy study will be conducted with 24 persons with PPA evaluating the adapted biographic-narrative approach Cope PPA. The efficacy study will be a mixed-methods randomised controlled trial with a waiting group, which is a suitable control (compared to standard care) for the inconsistent approach in the treatment of persons with PPA (Figure 1).

#### 2.3.1. Blinding

Due to the nature of the intervention, blinding of the investigators and participants is not possible. In ‘phase one’, independent raters are involved in the transcription and coding of the interviews. In ‘phase two’, external judgement assessments are evaluated by independent raters who can use the video recordings as the basis for the evaluation.

#### 2.3.2. ‘Phase One’: Focus-Group Interview

**Figure 2:**
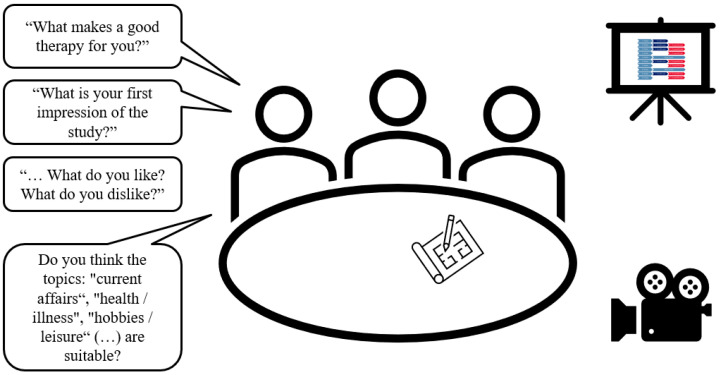
Focus group setting (designed by the author using PowerPoint images, Version 2401).

In the focus-group interview, the participants will be interviewed about their expectations/wishes for speech and language therapy and individual strategies for coping with their diagnosis (see Figure 2). Furthermore, the procedure and methods of narraktiv will be introduced. Persons with PPA will be asked to assess the extent to which methods and materials (e.g., use of pictograms or photographs) appear feasible and useful. The interviewer is a trained SLT (M.G.) who has some experience in conducting group discussions with aphasic participants. Repair behaviour such as repetition, revision/reformulation and cueing [42] are used to support participants’ communication. Turn-taking is organised by the interviewer. A text card saying “We listen to each other and let each other talk” is provided as a communication rule for the interview. If necessary, the participants are reminded of this agreement. The guidelines for the focus group interviews are provided in Additional File Supplementary Materials File S2.

#### 2.3.3. ‘Phase Two’: Efficacy Study

In ‘phase two’ of the study, participants will undergo the adapted biographic-narrative approach. Two groups will receive therapy immediately after randomisation, and participants enrolled in the waiting group will undergo therapy after an interval of 10 weeks. The first three individual therapy sessions will be based on the narrative interview format, which aims to elicit storytelling [43]. After an open initial stimulus (“Please, tell me your life story.”), participants will tell their life story without being interrupted. If necessary, the SLT will support the participant through phonological, semantic, and graphemic cues as well as paraphrasing or visual aids (pictures, pictograms, etc.). The main narrative could take up the entire first individual therapy session (90 min). It is also possible to continue the main narrative in the second to fifth individual therapy session. Basically, after the completion of the main narrative, there will be a phase of narrative-generating follow-up questions on only briefly mentioned topics (immanent questioning techniques, e.g., “You mentioned your childhood. What else comes to your mind about that time?”). After the immanent questions, new topics such as possible resources, self-theories, or ideas about the future will be made explicit (exmanent questioning techniques, e.g., “Above all your daughter helps you a lot. Why? What do you think?”). The fourth and fifth individual therapy sessions will be based on episodic interviews [44], which are more structured than narrative interviews. The episodic interview technique aims to elicit subjective definitions or arguments of personal relevance and narratives of subjectively meaningful life situations (e.g., “Are there situations that make you feel old? Please tell us.”) [44,45].

**Figure 3:**
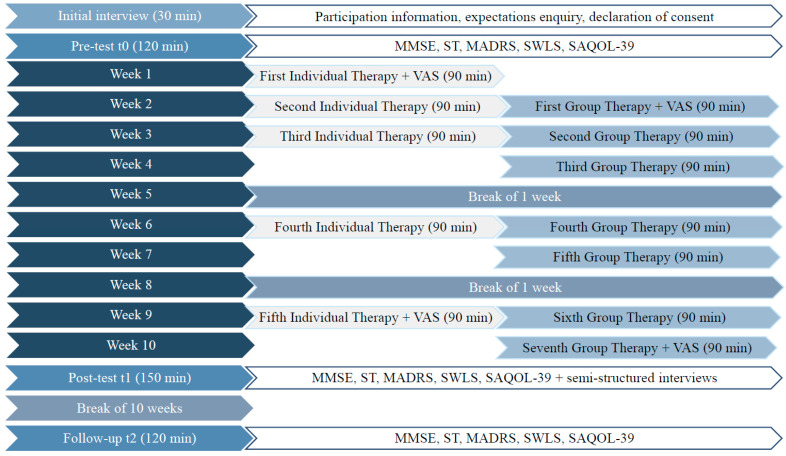
Efficacy study from a participant’s perspective. MMSE = Mini Mental-State Examination; ST = scenario test; MADRS = Montgomery–Asberg Depression Scale; SWLS = Satisfaction with Life Scale; SAQOL-39 = Stroke and Aphasia Quality of Life Scale-39; VAS = Visual Analog Scale.

From the second week onwards, group-therapy sessions (90 min each) will be conducted in addition to the individual-therapy sessions. Topics such as current events, hobbies, friends and family, health and illness will be addressed. After the third and fifth group-therapy sessions, a one-week break will be inserted to enable the integration of narrative experiences into daily life [19]. The entire course of the intervention from a participant’s perspective is shown in Figure 3.

#### 2.3.4. Randomisation

**Figure 4:**
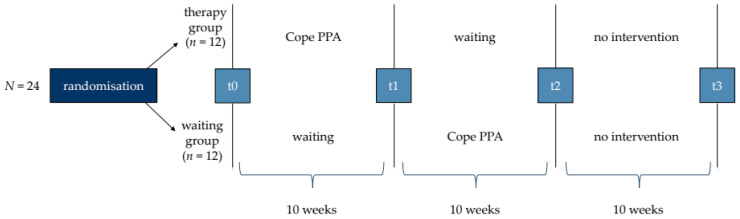
Procedure of ‘phase two’ containing four test points (t0, t1, t2, t3).

The division of the participants into groups is necessary for conducting the efficacy study and will be carried out as a random allocation by the deputy study director (M.G.) [46]. Since the therapy groups will consist of six participants, four blocks must be formed (N = 24). A permutation of the four blocks will be drawn at random, with the first half (*n* = 12) of the permutation being assigned to the therapy group and the second half (*n* = 12) assigned to the waiting group (see Figure 4). Each allocation thus applies to six consecutive participants. Groups will be randomised without balancing.

#### 2.3.5. Sample Size Justification

It can be assumed that the effects in persons with PPA are comparable to the effect sizes in persons with aphasia after stroke, as the impairment patterns are similar [42], and many approaches for the treatment of poststroke aphasia are successfully transferable to PPA [6,47]. Based on the results of Corsten et al. (2015), who reported an 85.19% improvement in Complaints and an 88.89% improvement in Burden score on the Aachen Life Quality Inventory [23] from baseline to follow-up, with an overall low to moderate effect size (d = 0.22 to 0.58) [19], an a priori power analysis was conducted using G*Power 3.1.7. This resulted in a total sample size of 24 for an estimated effect size of f = 0.275 (d = 0.55; α = 0.05; power = 0.8). It is assumed that with this sample size, effects can be detected in persons with PPA who are examined with the Stroke and Aphasia Quality of Life Scale-39 (SAQOL-39, [48]). It is possible that the effect sizes in persons with PPA are even greater if persons with mild depression are included; thus, a lower baseline score for QoL can be expected.

### 2.4. Measurements

All participants enrolled in ‘phase two’ will be assessed through self-assessments and external assessments at four test times (t0, t1, t2, t3) every 10 weeks.

#### 2.4.1. Measuring Instruments

The primary outcome of QoL will be measured quantitatively by using the SAQOL-39 [48]. The SAQOL-39 is a feasible, reliable and valid assessment of health-related QoL in persons with chronic poststroke aphasia [48]. There is an international consensus that this measurement tool is best-suited for assessing the QoL of persons with aphasia [49]. Furthermore, it has been used successfully in persons with PPA [50]. For the present study, the SAQOL-39 seemed more suitable than the Aachen Life Quality Inventory used by Corsten et al. (2015), as it has a higher psychometric quality in comparison.

As a secondary outcome, depression will be measured by using the MADRS [39]. With the intention of not assessing persons with PPA “to destruction” [51], the MADRS was also chosen because it is an indirect method. The MADRS is a reliable and valid instrument, with high values indicating higher levels of depression [39].

In addition, the Satisfaction With Life Scale (SWLS, [52]) will be used as a scale to measure global life satisfaction. The SWLS has been shown to have good psychometric properties, including high internal consistency, and is less influenced by positive affect [52]. As the SWLS comprises only five items, it can be carried out quickly, and so it is presumably not an additional burden for persons with PPA.

The MMSE [37] will be used to measure cognitive functioning. On the one hand, this is important to ensure the inclusion criteria, and on the other hand, the progression of PPA over the course of the study period will be monitored. In clinical and research settings, the MMSE is the most widely used short cognitive test. It is reliable and valid with little effect on practice [37].

The scenario test (ST, [53]) measures participants’ communicative abilities and consists of 18 items (e.g., shopping or visiting a doctor). Due to the facilitated role-plays, the process is very close to realistic everyday communication. Recent evidence suggests that this method is well applicable to persons with PPA [54].

The Visual Analog Scale (VAS, [55]) will be administered before and after the first and fifth individual therapy sessions as well as before and after the first and fifth group therapy sessions. In contrast to the VAMS, which was used in the study of Corsten et al. (2015), the VAS consists of a single horizontal line instead of a vertical line and is bipolar, similar to the preceding scales of Stern et al. (1997, [22]). The VAS is a widely accepted scale that was adapted for various clinical questions examining pain or the effects of interventions, with special reference to QoL. However, to the best of our knowledge, the VAS, according to Ushijima et al. (2006), has neither been validated nor used for the evaluation of mood in persons with primary progressive aphasia.

After each individual-therapy session and each group-therapy session, the SLT will fill out a fidelity checklist, which is intended to record how well the SLT adheres to the adapted biographic-narrative Cope PPA manual. The checklist contains the basic principles of the biographic-narrative approach (e.g., resource-oriented attitude/nondirective communicative behaviour). For the SLT, the fidelity checklist will serve as a self-reflection tool (see Additional Supplementary Materials File S4). All assessment appointments as well as all therapy sessions will be recorded by video to evaluate procedures such as the MADRS and the ST afterwards by an independent rater. Similar to the randomised controlled pilot study by Volkmer et al. (2023) [16,56], a random sample of 10% of the videos of the individual- and group-therapy sessions will be evaluated by an independent rater to assess fidelity too (see Additional Supplementary Materials File S5). Thus, the quality of the treatment will be recorded.

In addition to the quantitative evaluation, semistructured interviews will be conducted by an independent rater, with all participants and their caregivers asking for experiences during the intervention or results of participation. Participants’ perceptions will complement the quantitative evaluation. We will use field notes to capture the content of the interviews. An overview of all measurements is given in Table 1.

#### 2.4.2. Data Management

All personal information will remain confidential. The data will be stored at the University Hospital Mainz only. Only members of the research team (M.G., A.L.K., K.G., I.H.) will have access. Participants will be assigned a unique code (see “Randomisation” above), which will be on the assessment sheets, in the names of the video files, and in the analysis of documents and publications. The list of participant names and their unique codes will be kept in a locked cabinet in the rooms of the outpatient memory clinic.

#### 2.4.3. Data Analysis

Qualitative data analysis will be carried out using MAXQDA (VERBI Software, 1989–2021); quantitative data analysis will be carried out using the statistical software SPSS (version 27, IBM^®^) and RStudio (2023.09.1 Build 494).

The data from ‘phase one’ will be analysed via qualitative content analysis by Elo and Kyngäs (2008, [62]). The video material of the focus group interview will be transcribed according to the content semantic transcription by Dresing and Pehl (2018) by two SLTs [63]. The coding process will be carried out by two SLTs. Categories will be formed deductively. The category system for the analysis will contain categories such as ‘characteristics of good therapy’/‘Augmentative and Alternative Communication’/‘coping strategies’/‘opinion on therapy materials’.

The quantitative data of ‘phase two’ will be analysed descriptively and inferentially. Before the inferential analysis, the normal distribution will be checked in both groups using the Shapiro–Wilk test in conjunction with a Q–Q plot and values of skew and kurtosis [64]. For the primary outcome of QoL, analysis of variance (ANOVA) will be used to calculate the point and mean values (before–after and therapy-waiting group comparisons). In addition, the dependent structure of the data will be examined using the intraclass correlation coefficient (ICC). To analyse the individual cases, the critical difference between the test procedures used will be determined. Recruitment, participation, fidelity, and reasons for drop-outs or missing data will be documented using descriptive statistics.

Semistructured interviews of ‘phase two’ will also be transcribed according to the content semantic transcription by Dresing and Pehl (2018, [63]) and analysed according to the qualitative content analysis by Elo and Kyngäs (2008, [62]). The category system for the analysis will contain categories such as ‘general conditions’, ‘content’, ‘materials’, ‘evidence of improvements in QoL’, or ‘evidence of identity work’.

Quantitative analysis is performed as both a per-protocol and intention-to-treat analysis to maximise external and internal validity while minimising statistical bias to maximise external and internal validity while minimising statistical bias. Complete drop-outs will be treated differently from missing values (e.g., due to an individual’s absence in individual or group therapy sessions in which a VAS was conducted). In these individual cases, imputation is considered. There are no plans to promote the retention of participants or intentions to follow-up in the case of individuals who drop out or deviate from the intervention protocol. There is no interim analysis planned.

#### 2.4.4. Dissemination

Due to the fact that the research project is part of a cumulative doctorate, several publications, lectures and poster presentations are planned. Participants in the study will be informed about publications. Content will be made available in plain language (e.g., through ‘Lay Abstracts’).

#### 2.4.5. Criteria for Success

The criteria for success of ‘phase two’ are defined as follows:

1. Significant improvement in SAQOL-39 score with a medium effect size from pre- to post-intervention.
2. Improvements in SAQOL-39 score between t0 and t1 are significantly greater in the therapy group than in the waiting group.
3. In the semistructured interviews, the participants report generally positive perceptions about the acceptability of the intervention.
4. The fidelity rate is at least 80% in the random sample evaluated by independent rater.
5. Stable values or improvements on the MADRS.
6. Stable values or improvements on the VAS.

#### 2.4.6. Assessment and Management of Risk

Both through participation in the focus group interview and through participation in the group therapy sessions, participants might meet other persons with PPA who are more advanced in the course of the disease than they are themselves. This confrontation with other participants’ symptomatology could initially increase awareness of their own disorder. However, most of the research on this topic suggests that persons with PPA generally experience interactions with peers in a positive way [13,65]. Our own clinical experience with previous completed studies supports these findings.

The confrontation with one’s personal history in the biographic-narrative approach can also stimulate negative memories. Nevertheless, Corsten et al. (2015) provided empirical evidence that participants usually find a positive way of dealing with the experience on their own. The SLT will pay attention to spontaneous reactions of the participants, such as meta-linguistic comments, changes in facial expression, increased sighing, or zero reactions. Signs of excessive demands or discomfort will be documented and addressed in individual discussions with the participants concerned. If the deputy study director (M.G.) or the clinicians involved (I.H., K.G.) come to the conclusion that the continuation of the therapy would harm the participants, the therapy will be stopped immediately. There is no audit for the planned study.

## 3. Discussion

To the best of our knowledge, this is the first study including a randomised controlled trial design using a biographic-narrative approach in persons with PPA. Although reminiscence interventions have been used successfully for the treatment of dementia in general [66], only one case study known to us has mentioned the use of the biographic-narrative approach narraktiv in a person with PPA, and the effects were not described [32]. The planned sample size of 24 participants in the present study is also special for a rare disease.

The limitations of this study include the fact that the primary outcome of the study, QoL, is not well defined and is difficult to measure in persons with PPA. Formal assessments that have been used with PPA, such as the Aphasia Impact Questionnaire 21 [16,67], have not yet been translated into or validated in the German language. Thus, the SAQOL-39 will be used, an instrument that is internationally recognised and counted among the core outcome sets [49]. However, the use of an assessment that is standardised for persons with poststroke aphasia and therefore contains items that are not directly related to the symptoms of PPA (e.g., items about mobility) remains a limitation of our study that has arisen due to the lack of German-language versions of measures tailored for persons with PPA [7]. To counteract this limitation, our study additionally utilises semi-structured interviews as qualitative assessments with the aim of gaining additional insights into the impact of our intervention. Influencing and disorder-specific factors should be identified and explored.

It is important to note that the expected improvements in QoL and mood are only potential outcomes. However, there are also challenges that could occur during intervention, e.g., it is possible that cognitive deficits impair storytelling and that the stimulation of lost memories can lead to feelings of frustration [26]. While narraktiv generates improvements in the QoL of persons with chronic aphasia, we must consider that persons with a progressive disease will be targeted in our study. By decreasing awareness of deficits in the course of PPA, it is conceivable that an improvement in QoL may not (only) be due to the intervention. Due to these circumstances, communicative–pragmatic and cognitive functions will be assessed.

Another limitation of the planned waiting group control design is that it cannot be ruled out that individual effects may result from other circumstances that arise during the waiting period [68]. For ethical reasons, participants are permitted to take advantage of other therapies in addition to the biographic-narrative approach of the present study. Concurrent use of, e.g., outpatient speech and language therapy could influence the results of the intervention and carry a risk of bias. As our programme is an intensive therapy (up to twice weekly sessions, 90 min each), it is unlikely that additional influences will have a large effect.

There are many reasons why a biographic-narrative approach could be quite efficient in persons with PPA. First, persons with poststroke aphasia and persons with PPA face similar biographic disruptions due to their speech disorders, which makes the transfer of such approaches reliable. Furthermore, the fact that autobiographical knowledge is retrievable for a long period of time during the course of PPA makes biography a suitable approach [69]. The fact that PPA is such a rare condition might strengthen the influence of a conducted group setting. The findings of the semistructured interviews, in which persons with PPA report what they have experienced as valuable or what they take away from the therapy, could point the way here.

Overall, this is the first study to investigate the efficacy of the biographic-narrative approach described by Corsten et al. (2015) in persons with PPA. If the biographic-narrative approach Cope PPA is effective, it will contribute to an enormous gain in knowledge in the field of research for persons with PPA.

## Figures and Tables

**Table 1:** Measuring instruments, aim and time of measurement.

Measuring Instruments	Aim of Measuring	Time of Measuring
Questionnaires (filled out by participants)		
39-Item Version of the Stroke and Aphasia Quality of Life Scale (SAQOL-39, [48,57])	quality of life	t0, t1, t2, t3
Satisfaction with Life Scale (SWLS, [52,58])	satisfaction with life	t0, t1, t2, t3
Assessments (conducted by SLT, evaluated by independent rater)		
Montgomery–Asberg Depression Scale (MADRS, [39,59])	depression	t0, t1, t2, t3
Mini Mental-State Examination (MMSE, [37,60])	cognitive status	t0, t1, t2, t3
Scenario test (ST, [53,61])	communicative–pragmatic function	t0, t1, t2, t3
Visual Analog Scale (VAS, [55])	mood	before and after the first and fifth individual therapy sessions; before and after the first and fifth group therapy sessions
Interviews (conducted by independent rater with participants and caregiver)		
Semistructured interviews	views and satisfaction with the current therapy; effectiveness of the intervention	after intervention
Checklists (filled out by SLT)		
Fidelity checklist	self-reflection according to adherence to the biographic-narrative approach	after every individual and every group therapy session
Checklists (filled out by independent rater)		
Fidelity checklist	recording of quality and evaluation of adherence in a random sample of 10% of video recordings of all therapy sessions	at the end of the study

## Data Availability

The data are not publicly available due to privacy and ethical restrictions.

## Supplementary Materials

The following supporting information can be downloaded at: https://www.mdpi.com/article/10.3390/brainsci14070692/s1, Additional Supplementary Materials File S1: SPIRIT 2013 Checklist; Additional Supplementary Materials File S2: Interview-Guide; Additional Supplementary Materials File S3: Fidelity checklist (self-rating); Additional Supplementary Materials File S4: Fidelity checklist (independent rating); Additional Supplementary Materials File S5: Consent form—Focus group interview (translated from German to English); Additional Supplementary Materials File S6: Consent form—Efficacy study (translated from German to English).
