# Supplementary material for: Cope PPA—Adaptation of the Biographic-Narrative Approach for Persons with Primary Progressive Aphasia: Protocol for Clinical Trial Design
Source: Brain Sci. 2024 Jul 11;14(7):692. doi: 10.3390/brainsci14070692 (PMC11274457; doi:10.3390/brainsci14070692)
Supplement: Supplementary file 1 [file brainsci-14-00692-s001.zip › Supplementary Materials File S2.pdf]

Additional Table: Focus group interview-Guide (Pre-intervention) for five individuals with PPA

Questions	***Enquiries***
(greeting participants, getting to know each other, switching on the camera)<br>Thank you very much for agreeing to take part in today's interview. (Maybe: You have already taken part in one of our group therapies. Maybe: you have an outpatient speech and language therapist. So, you are real therapy experts.)	
(1) First of all, I would like to know: What makes a good therapy for you?	What do you think a therapist should be like?<br>Do you like it when your therapist offers to help you speak?<br>Do you like it when you are given tasks to do at home?
(2) What would you need to tell about your life story?	For example, do you sometimes write things down?<br>Do you use gestures?<br>Do you write down things you want to say?<br>Do you use a communication book?<br>Is there a technical aid that you use?<br>*(if necessary, show "ACC tools" as an example)*
(3) What helps you to deal with your disease?	*(provide pictograms so that AAC can be used for zero response: family, friends, personality, medical staff, sport, other hobbies, faith/religion, ...)*<br>Are there experiences from your past that help you to deal with the illness today?
Presentation narraktiv in easy language (Powerpoint slides + e.g. You know we want to research a therapy concept. The therapy concept consists of... The aim is to make people feel better after the therapy...)	
(4) What is your first impression of the *narraktiv* approach?	What do you like?<br>What do you dislike?<br>What is particularly important to you?
(5) You have just heard that there is an activity diary in the *narraktiv* programme.<br><br>Do you think it is helpful to keep a record of what you have experienced during the therapy - like in a diary?	Why do you think so?<br><br>(If necessary, what would you need to record what you have experienced? Would your spouse need to support you in this?)
(6) You have heard that there are communication rules in the *narraktiv* programme.<br><br>Do you think the communication rules written down here are suitable?	Why do you think so?<br><br>(*show Power Point slide*) Which rules are important to you / would you add?<br><br>Do you think communication rules should be defined by the group members?
(7) You have heard that expectations are asked at the beginning of the *narraktiv* programme?<br><br>Do you think it is helpful for therapy if expectations are discussed in advance?	Why do you think so?
(8) You have gained an impression of the topics addressed in the *narraktiv* programme.	Why do you think so?<br><br>Which topics would you add?

Do you think the topics: "current affairs" / "health / illness", "hobbies / leisure", "career / retirement", "social relationships / spirituality" are suitable?	
(9) You have seen the material used in the *narraktiv* programme to help people tell their life stories.<br><br>Do you think that photos / pictograms / a timeline would support the telling of your life story? (h*ave examples ready*)	Why do you think so?<br><br>Can you think of any other non-linguistic methods that you would like to use?
(10) We have already talked about materials.<br><br>Do you think it would be good to use methods from art or music therapy to support the biography work?<br><br>(*Give & show examples*)	Why do you think so?<br><br>Can you think of any other non-linguistic methods that you would like to use?
(11) It might be helpful to have a repository for all the material.<br><br>In other studies, people have made story books or used memory boxes (have examples ready)<br><br>Do you think such a story book or memory box is useful so that you can take something home with you at the end of therapy?	Why do you think so?<br><br>Can you think of anything else here?
(12) You have heard that the *narraktiv* programme offers the opportunity to give verbal feedback at the end of each session.<br><br>Do you think this form of feedback is useful?	Why do you think so?<br><br>What form of feedback can you think of here?
We have already reached the end of time and unfortunately have to come to a conclusion at this point... *(thanks; renewed information about what happens to the data; reference to planned lay report; camera off)*	

Additional Table 1: Interview-Guide of the semistructured interview (Post-intervention) for participants

Questions	***Enquiries***
(1) How did you experience the intervention?	What did you think of the individual and group therapies?<br><br>What did you think?<br>- Activity diary<br>- Communication rules<br>- Expectation survey<br>- Topics<br>- Material<br>- Non-linguistic methods

	- Story book / memory box<br>- Feedback<br><br>How did you find the time frame? (duration / frequency)
(2) What could you take away from your participation?	Was there anything that particularly helped you?<br>*If yes:* What?
(3) Has anything changed as a result of your participation? *If yes:* What?	Has participation had an impact on your view of:<br>- your past?<br>- your life?<br>- your future?<br>*If yes:* How?

Additional Table 2: Interview-Guide of the semi-structured interview (Post-intervention) for caregivers

Questions	***Enquiries***
(1) What do you think how your partner experienced the intervention?	How do you think he/she experienced individual and group therapies?<br><br>How did you - as a relative - experience the time frame? (duration / frequency)
(2) What do you think has he / she taken away from participation?	Was there anything that particularly helped him / her?<br>*If yes:* What?
(3) Has anything changed for you personally through the participation of your partner? *If yes:* What?	*If yes:* What has changed?
