# Supplementary material for: Cope PPA—Adaptation of the Biographic-Narrative Approach for Persons with Primary Progressive Aphasia: Protocol for Clinical Trial Design
Source: Brain Sci. 2024 Jul 11;14(7):692. doi: 10.3390/brainsci14070692 (PMC11274457; doi:10.3390/brainsci14070692)
Supplement: Supplementary file 1 [file brainsci-14-00692-s001.zip › Supplementary Materials File S4.pdf]

Additional Table: Independent-rating checklist

**Observable behaviours**	Fully agree (4)	Partly agree (3)	Partly disagree (2)	Fully disagree (1)	Not applicable (0)	Comments
1. The therapist did her best to facilitate the communication of the person with aphasia (e.g. by paraphrasing / using sounds / using pictures, pictograms or written language). During the main narrative, however, she held back more so as not to interrupt the narrative flow.						
2. At the end of each session the therapist asked the participant to give feedback and reflect on their own mood.						
3. The therapist focussed on non-judgemental storytelling.						
4. The therapist only interrupted the participant if it was unavoidable (e.g. because she did not understand something).						
5. The therapist did not correct the participant linguistically.						
6. The therapist could stand breaks.						
7. The therapist put her own opinion aside and did not provide any solutions.						
8. The therapist picked up on disturbances (noise etc.) and only then continued the conversation.						
9. The therapist took up statements made by the participant (e.g. by repeating / paraphrasing) and during the main narrative and in the case of immanent questions she has only dealt with those contents that were mentioned by the participant.						
10. The content of the session was customised to the individual participant (e.g. by selecting questions from the guide).						
11. The participant was encouraged by the therapists questions to report biographical content (specific content such as hobbies).						
12. The therapist asked exmanent questions in a way that emphasised the participant's resources.<br><br>**! This item is only applicable from the point at which exmanent questions are used. Before that, it should be disregarded in the assessment!**						
13. The therapist addressed all three levels of time (past, present and future) today.<br><br>**! This item is only applicable when new topics have been introduced with the help of external questions. Before that, it should be disregarded in the assessment!**						
14. The therapist introduced new topics (e.g. health / illness) in a non-judgemental way so that they could be evaluated individually by the participant.						

**! This item is only applicable once the terms health / illness have been introduced. Before that, it should be neglected in the assessment!**						
15. The expectations and wishes of the participant were taken up by the entire group with a basic attitude of acceptance and without judgement.<br><br>**! This item is only applicable for the group sessions and can be neglected in the evaluation of the individual sessions!**						

Assessment of observable behaviour (max. 44 to 60 points)
If items 12-15 are applicable, the total score should be divided by 60.
If three of the items 12-15 are applicable, the total score should be divided by 56.
If two of the items 12-15 are applicable, divide the total score by 52.
If only one of the items 12-15 is applicable, divide the total score by 48.
If none of the items 12-15 are applicable, divide the total score by 44.

The proportional total value (< 1) can be multiplied by 100.
The result describes the percentage of adherence to the *narraktiv* guideline.

An example of the evaluation of a group session after the introduction of the terms health / illness and new topics:
(40 / 60) x 100 = 66,7 %
