# Supplementary material for: Cope PPA—Adaptation of the Biographic-Narrative Approach for Persons with Primary Progressive Aphasia: Protocol for Clinical Trial Design
Source: Brain Sci. 2024 Jul 11;14(7):692. doi: 10.3390/brainsci14070692 (PMC11274457; doi:10.3390/brainsci14070692)
Supplement: Supplementary file 1 [file brainsci-14-00692-s001.zip › Supplementary Materials File S5.pdf]

Mirjam Gauch (M.Sc.)
Deputy Director of Studies and Speech Therapist
Department of Psychiatry and Psychotherapy
University Hospital Mainz
Untere Zahlbacher Straße 8
55131 Mainz

Participant information and informed consent for the study:
Biography work for persons with PPA
- Focus group interview –

Full title:
The influence of biography work according to the narraktiv protocol by Corsten et al. (2015) on the quality of life of persons with primary progressive aphasia

Dear participants,

with this letter we would like to inform you about the above-mentioned study at our clinic and ask about your interest in **voluntary** participation in the study. Please read this information letter carefully. Your doctor will also talk to you directly about this study. Please ask him/her if there is anything you do not understand or if there is anything else you would like to know.
The study was ethically reviewed by the Ethics Committee of the State of Rhineland-Palatinate and approved on 18 January 2024.

Aim of the study

Biography work, i.e. telling stories from one's own life, is used in the therapy of persons with aphasia after a stroke and in the therapy of dementia syndromes in Alzheimer's disease. Individual case studies have shown that persons with primary progressive aphasia (PPA) can benefit from biography work. Larger-scale studies investigating biography work in PPA and a standardised procedure do not yet exist. The biographic-narrative approach is one way in which biography work can be carried out by speech and language therapists. We know from studies that this approach can improve quality of life and mood for persons with aphasia after stroke. In this first study phase, we want to find out to what extent the biographic-narrative approach needs to be adapted for persons with PPA. The adaptations will form the basis for a larger-scale efficacy study in our second study phase.

Study realisation

Before you are included in the study, you will have an information session (15 minutes) with a doctor from the outpatient memory clinic and the speech therapist who will conduct the focus group interview. You will then receive all the information you need to take part. Your written declaration of consent is a prerequisite for participation. Following the information meeting, you will be offered an appointment for the focus group interview. In addition to you, four other persons with PPA will take part in this interview (120 minutes). In the interview, you can discuss your expectations of the biography work with other persons affected and give feedback on the content and methods of the planned study. By taking part, you can help to ensure that individual and group therapies are even better adapted to the needs of persons with PPA.

Possible benefits for you

By participating in the study, you will have the opportunity to help determine what future care could look like. In this way, you can contribute to the care of persons with PPA and improve your own situation as well as the situation of others affected. If you are interested in participating in the study after the interview, you can also take part in the individual and group therapies.

Mirjam Gauch (M.Sc.)
Deputy Director of Studies and Speech Therapist
Department of Psychiatry and Psychotherapy
University Hospital Mainz
Untere Zahlbacher Straße 8
55131 Mainz

In this case, you would benefit directly from the feedback you provided to the research team during the focus group interview.

Risks and burdens

By taking part in the focus group interview, you will meet other persons with PPA. They may be more advanced in the course of the disease than you are. The confrontation with other persons' problems could be stressful for you. If you feel stressed during the two-hour interview, be sure to report this to the interviewer. You have the option of cancelling the interview at any time if you wish. If you notice increased stress after the interview, the interviewer and other medical members of the research team will be available to help you. However, studies show that an exchange with other affected persons is generally experienced as positive despite such confrontations.

Insurance cover

No separate insurances are planned. The insurance policies of the University Hospital Mainz apply.

Voluntariness and cancellation option

Participation in this study is **voluntary**. In addition to the written information, you will be informed verbally. If you do not understand something, please ask the doctor giving you the information. You will then have time to decide if you want to take part. If you decide to take part, please sign the declaration of consent.

You may **revoke** your consent at any time (orally, in writing or in text form) and object to the further processing of your data. You do not need to give any reasons for this. This will not affect your medical treatment or your relationship with your doctor. If you stop participating in the study, your personal and pseudonymised data will be deleted. Your cancellation will only take effect from the time the declaration is received by the University Hospital Mainz. It has no retroactive effect, i.e. the processing of your data up to this point in time remains lawful. After deletion of the coding list, only anonymous data is available, so that retroactive deletion of the data is no longer possible.

Data collection

We guarantee compliance with data protection regulations. Your personal data will be always treated confidentially. The data will be stored at the University Hospital Mainz.

The focus group interview will be recorded on video. The video is used to analyse the results more precisely. The video will be deleted immediately after the evaluation. The results of the evaluation are stored in pseudonymised form.
Pseudonymised means that your personal data (e.g. name, date of birth and address) is replaced by a value-neutral code (e.g. XYZ01). Your study data can be assigned via this code without your personal data being accessible. Access to the coding list and thus to potential reverse coding lies solely with the employees of University Hospital Mainz who are involved in the study. The coding list will be deleted immediately after the end of the project. Consultation with the data protection officer of the University Hospital Mainz has taken place.

Mirjam Gauch (M.Sc.)
Deputy Director of Studies and Speech Therapist
Department of Psychiatry and Psychotherapy
University Hospital Mainz
Untere Zahlbacher Straße 8
55131 Mainz

Inclusion of the requirements of the GDPR

In accordance with the European General Data Protection Regulation (GDPR), we inform you below about your rights in relation to the processing of your personal data:

I. Type of data:

The data that we process from you is personal data in accordance with Art. 4 No. 1 GDPR or special categories of personal data in the form of health data in accordance with Art. 4 No. 15 GDPR.

We process your personal data (name, address, date and place of birth) as well as the following health data as part of the study
- Socio-demographic data (gender, age, level of education, multilingualism, relationship status)
- Health-related data (diagnoses, previous drug and non-drug therapies)
- Video recordings of the examination appointments

II Purpose of processing:

Your personal data will be processed exclusively for the study purpose described above. Your data will not be processed beyond the purpose of the study.

III Legal basis:

The legal basis for the personal data processed by you as part of the above-mentioned study is your declaration of consent in accordance with Art. 7 and Art. 9 para. 2 lit. a) GDPR and § 37 para. 1 of the Rhineland-Palatinate State Hospital Act (LKG RLP).

IV. Recipients/categories of recipients:

The data will only be passed on to third parties (persons who are not involved in the above-mentioned study) or published in anonymised form. The results will be published as part of a doctoral thesis. In addition, up to four publications on this study are planned in scientific journals.

V. Storage period and deletion:

The study-related data will be deleted after the study objective has been achieved, but at the latest after 10 years or in the event of withdrawal of consent, unless longer retention periods are required by law.

VI Rights of data subjects:

You have the following rights with regard to your data:

Right to information in accordance with Article 15 GDPR:

You have the right to request information about whether and which of your personal data is processed by us. The right to information can be restricted in accordance with

Mirjam Gauch (M.Sc.)
Deputy Director of Studies and Speech Therapist
Department of Psychiatry and Psychotherapy
University Hospital Mainz
Untere Zahlbacher Straße 8
55131 Mainz

Section 27 (2) BDSG, i.e. if data is not processed automatically - for example, only on paper.

Right to rectification in accordance with Articles 16, 19 GDPR:
You have the right to have incorrect personal data concerning you rectified. The right to rectification may be restricted in accordance with Section 27 (2) BDSG.

Right to erasure in accordance with Articles 17, 19 GDPR:
You have the right to request the erasure of personal data concerning you. If you request the erasure of your data, all personal data relating to you will be erased.

Right to restriction of processing in accordance with Articles 18, 19 GDPR:
You have the right to restrict the processing of personal data concerning you. The right to restriction of processing may also be restricted within the meaning of Section 27 (2) BDSG.

Right to data portability in accordance with Art. 20 GDPR:
You have the right to receive the personal data concerning you that you have provided to the controller for the study. This means that you can request that this data be transferred either to you or, if technically possible, to another organisation named by you.

Right to object pursuant to Art. 21 GDPR:
You have the right to object to the processing of personal data concerning you. This right may be restricted in accordance with Section 27 (2) BDSG.

VII Automated decision-making
Your personal data is not subject to decisions based solely on automated processing (e.g. profiling).

VIII. Consequences of non-provision
The provision of personal data is not required by law or contract, nor is it necessary for the conclusion of a contract. The provision of the data does not constitute an obligation. Failure to provide the data has no consequences.

IX. Person responsible
Responsible for processing of data:
Hospital of Johannes Gutenberg University Mainz
represented by the Executive Board
Langenbeckstraße 1, 55131 Mainz
Telephone: 06131 17-0
Website: http://www.unimedizin-mainz.de/

Mirjam Gauch (M.Sc.)
Deputy Director of Studies and Speech Therapist
Department of Psychiatry and Psychotherapy
University Hospital Mainz
Untere Zahlbacher Straße 8
55131 Mainz

You have the right to lodge a complaint with a supervisory authority of your choice. The competent supervisory authority for the University Hospital Mainz is the State Commissioner for Data Protection and Freedom of Information Rhineland-Palatinate:

Postfach 30 40, 55020 Mainz
Hintere Bleiche 34, 55116 Mainz
Tel.: +49 (0) 6131 208-2449
Fax: +49 (0) 6131 208-2497
Email: poststelle@datenschutz.rlp.de
https://www.datenschutz.rlp.de

Contact details of the Data Protection Officer of the University Hospital Mainz:
Langenbeckstraße 1
55131 Mainz
Tel.: 06131/17-0
Email: datenschutz@unimedizin-mainz.de

Contact information

If you have any questions or wish to assert your rights as a data subject, please do not hesitate to contact me:

Mirjam Gauch
Logopädin, M.Sc. (Healthcare and Nursing)
Department of Psychiatry and Psychotherapy
University Hospital Mainz
Untere Zahlbacher Straße 8, 55131 Mainz
Tel.: 06131/17-2474
E-Mail: mirjam.gauch@unimedizin-mainz.de

Yours sincerely,

………………………………… ……………………………………………………

Place, date Name, signature (Deputy Director of Studies)

Mirjam Gauch (M.Sc.)
Deputy Director of Studies and Speech Therapist
Department of Psychiatry and Psychotherapy
University Hospital Mainz
Untere Zahlbacher Straße 8
55131 Mainz

Participant information and informed consent for the study:
Biography work for persons with PPA

Full title:
The influence of biography work according to the narraktiv protocol by Corsten et al. (2015) on the quality of life of persons with primary progressive aphasia

I hereby declare,

First name and surname: ………………………………………………………………………,
Please complete in block letters.

Date of birth: …………………………………………………………………………..............,

Participant Code: …………………………………………………………………….............,
(Pseudonym, will be entered by the study director)

I am willing to participate voluntarily in the above-mentioned study. I was informed in a personal interview by
Mr/Mrs/Ms
....
(Name of the study doctor)

I was informed in detail and clearly about the nature, significance, risks and implications of the study. I had the opportunity to have a counselling interview. All my questions were answered satisfactorily and I can ask new questions at any time. I have also read and understood the text of the information leaflet.
I had enough time to make up my mind.
I have received, read and understood a copy of the information sheet and the declaration of consent. The original of the signed declaration of consent will remain at the test centre.
I declare that I am voluntarily willing to participate in the scientific study. I have been comprehensively informed about the processing of my personal data in accordance with Art. 13 GDPR.

PRIVACY POLICY:
I have understood and expressly agree

1. **that my personal data required for the purpose of the above-mentioned study will be collected by the study doctor and recorded and processed in pseudonymised form, including on electronic data carriers;**
2. **that the study results will be published in an anonymous form that does not allow any conclusions to be drawn about my person;**

UNIVERSITÄTSmedizin. MAINZ

Mirjam Gauch (M.Sc.)
Deputy Director of Studies and Speech Therapist
Department of Psychiatry and Psychotherapy
University Hospital Mainz
Untere Zahlbacher Straße 8
55131 Mainz

3. **that my data will only be processed for the above-mentioned purposes and only by study staff or the named recipients or categories of recipients;**
4. **that a video recording of the focus group interview will be made in which I can be recognised and which serves to adapt the biographic-narrative approach. The video recordings will be deleted immediately after evaluation.**

I can revoke my consent to participate in the study at any time and without giving reasons (verbally, in writing or in text form) and object to the further processing of my data and samples without incurring any disadvantages. In this case, my personal data will be deleted. My revocation will only take effect from the time of receipt of the declaration by the University Hospital Mainz. It has no retroactive effect, i.e. the processing of my data up to this point in time remains lawful.

○ **I would like to be informed about the results following the study.**

………
Place, date Signature of the study doctor

………
Place, date Signature of the study participant

if applicable

………
Place, date Signature of the legal representative

I hereby declare,

Name of the study doctor ………………………………………………………

of the study participant ....

to have informed verbally and in writing about the nature, significance and risks of the above-mentioned study, to have answered all questions and to have been given a copy of the study information and the informed consent form.

………
Place, date Signature of the study doctor
