# Supplementary material for: Cope PPA—Adaptation of the Biographic-Narrative Approach for Persons with Primary Progressive Aphasia: Protocol for Clinical Trial Design
Source: Brain Sci. 2024 Jul 11;14(7):692. doi: 10.3390/brainsci14070692 (PMC11274457; doi:10.3390/brainsci14070692)
Supplement: Supplementary file 1 [file brainsci-14-00692-s001.zip › Supplementary Materials File S6.pdf]

Mirjam Gauch (M.Sc.)
Deputy Director of Studies and Speech Therapist
Department of Psychiatry and Psychotherapy
University Hospital Mainz
Untere Zahlbacher Straße 8
55131 Mainz

Participant information and declaration of consent for the study: Biography work for persons with PPA - Efficacy study -

Full title:
The influence of biography work according to the narraktiv protocol by Corsten et al. (2015) on the quality of life of persons with primary progressive aphasia

Dear participants,

With this letter we would like to inform you about the above-mentioned study at our clinic and ask for your interest in **voluntary** participation in the study. Please read this information letter carefully. Your doctor will also talk to you directly about this study. Please ask him/her if there is anything you do not understand or if there is anything else you would like to know.
The study was ethically reviewed by the Ethics Committee of the state of Rhineland-Palatinate and approved on 18 January 2024.

Aim of the study

Biography work, i.e. telling stories from one's own life, is used in the therapy of persons with aphasia after a stroke and in the therapy of dementia syndromes in Alzheimer's disease. Individual case studies have shown that persons with primary progressive aphasia (PPA) can benefit from biography work. Larger-scale studies investigating biography work in PPA and a standardised procedure do not yet exist. The biographic-narrative approach is one way in which biography work can be carried out by speech and language therapists. We know from studies that this approach can improve quality of life and mood for persons with aphasia after stroke. In an initial study phase, we identified how the biographic-narrative approach needs to be adapted for persons with PPA. The extent to which the approach we have adapted can improve the quality of life and mood of persons with PPA will be determined in this second phase of the study.

Study realisation

Before you are included in the study, you will have an information session (30 minutes) with a doctor from the outpatient memory clinic and the speech therapist, who will also carry out the biography work afterwards. You will receive all the information you need to participate and can express your expectations. Your written declaration of consent is a prerequisite for participation in the study. A pre-test (120 minutes) will then take place at the start of the study. Several tests will be used to measure your cognitive and linguistic abilities as well as your mental health and quality of life. These tests are carried out four times at 10-week intervals throughout the course of the programme. These appointments are for monitoring and quality assurance purposes. They take 120 minutes each. After the pre-test, the intervention consists of five individual therapies with a speech therapist and seven group therapies (90 minutes each) with two speech therapists. The group consists of six participants and is led by the speech therapist, who you will already know from the individual therapies, and a second speech therapist who provides support in the group therapies. The research project is a controlled, randomised study in which the effect of the therapy is compared with standard care. If you are already receiving speech therapy, you can continue to do so during the study. Participation in the study is an additional

Mirjam Gauch (M.Sc.)
Deputy Director of Studies and Speech Therapist
Department of Psychiatry and Psychotherapy
University Hospital Mainz
Untere Zahlbacher Straße 8
55131 Mainz

offer. Randomised means that once you are enrolled in the trial, you will be randomly assigned to either receive the therapy immediately or to continue treatment as usual and receive the therapy later.
Depending on which group you are assigned to, it is possible that you will have to wait a certain amount of time (around 10 weeks) after the pre-test before starting therapy (beginning with the first individual therapy session). There are methodological reasons for this. This procedure is known as the "waiting control group". At the end of the study, you will have received the same therapy as the participants in the other group. The therapy takes place over a period of ten weeks. At the end of the first and last individual therapy sessions and at the end of the first and last group therapy sessions, your mood will be assessed using a questionnaire. At the end of the therapy, we would like to hear your opinion on the individual and group therapies. For this reason, interviews (30 minutes each) are planned with you and your care partner. The appointments will all take place in the outpatient memory clinic at the Department of Psychiatry and Psychotherapy of the University Hospital in Mainz.

Possible benefits for you

We expect that the quality of life and mood of you as a participant will improve because of the therapy. These are the effects that have been observed in the studies for persons with aphasia after stroke. In addition, studies indicate that symptoms such as depression could be improved by therapies such as the one planned. We therefore expect that participation in the study may be of benefit to you. If these expected effects do not materialise, we know that group therapy can have a positive effect on the self-confidence of persons with PPA and that the group feeling that develops in such therapy can be a positive experience.

Risks and burdens

By joining a group, you may get to know other persons who are further along in their disease than you are. This confrontation with other persons' problems could be stressful under certain circumstances. However, studies show that group therapies are experienced as positive despite such confrontations. In the planned study, you will have the opportunity to talk about your life. Due to the language difficulties associated with PPA, you may find it difficult to talk. In such cases, you will be supported by the therapist. If you wish, she will offer you assistance so that you can succeed in telling the story. Nevertheless, you may occasionally find it frustrating, or you may remember sad events from your life. We will always enquire about how you are doing and can react to negative feelings. In such cases, it would be possible to initiate a consultation with a specialist of our clinic at any time.

Insurance cover

No separate insurances are planned. The insurance policies of the University Hospital Mainz apply.

Voluntariness and cancellation option

Participation in this study is **voluntary**. In addition to the written information, you will be informed verbally. Ask the doctor giving the information if there is anything you do not understand. You will then have time to decide if you want to take part. If you decide to take part, please sign the declaration of consent.

Mirjam Gauch (M.Sc.)
Deputy Director of Studies and Speech Therapist
Department of Psychiatry and Psychotherapy
University Hospital Mainz
Untere Zahlbacher Straße 8
55131 Mainz

You may **revoke** your consent at any time (orally, in writing or in text form) and object to the further processing of your data. You do not need to give any reasons for this. This will not affect your medical treatment or your relationship with your doctor. If you stop participating in the study, your personal and pseudonymised data will be deleted. Your cancellation will only take effect from the time the declaration is received by the University Hospital Mainz. It has no retroactive effect, i.e. the processing of your data up to this point in time remains lawful. After deletion of the coding list, only anonymous data is available, so that retroactive deletion of the data is no longer possible.

Data collection

We guarantee compliance with data protection regulations. Your personal data will be always treated confidentially. The data will be stored at the University Hospital Mainz.
Both the examination appointments and individual and group therapies are recorded on video. The videos are used to analyse the results even more precisely. The videos are deleted immediately after evaluation. The results of the evaluation are stored in **pseudonymised** form. Short tests and questionnaires are also carried out with you as part of the test appointments.

Pseudonymised means that your personal data (e.g. name, date of birth and address) is replaced by a value-neutral code (e.g. XYZ01). Your study data can be assigned via this code without your personal data being accessible. Access to the coding list and thus to potential reverse coding lies solely with the employees of the University Hospital Mainz who are involved in the study. The coding list will be deleted immediately after the end of the project. Consultation with the data protection officer of the University Hospital Mainz has taken place.

Inclusion of the requirements of the GDPR:

In accordance with the European General Data Protection Regulation (GDPR), we inform you below about your rights in relation to the processing of your personal data:

I. Type of data:
The data that we process from you is personal data in accordance with Art. 4 No. 1 GDPR or special categories of personal data in the form of health data in accordance with Art. 4 No. 15 GDPR.

We process your personal data (name, address, date and place of birth) as well as the following health data as part of the study:

- Socio-demographic data (gender, age, level of education, multilingualism, relationship status)
- Health-related data (diagnoses, previous drug and non-drug therapies)
- Video recordings of the examination appointments
- Results of questionnaires and checklists used (MMST, ST, MADRS, SWLS, SAQOL-39, VAS)

II. purpose of processing:

UNIVERSITÄTSmedizin. MAINZ

Mirjam Gauch (M.Sc.)
Deputy Director of Studies and Speech Therapist
Department of Psychiatry and Psychotherapy
University Hospital Mainz
Untere Zahlbacher Straße 8
55131 Mainz

Your personal data will only be processed for the purpose of the study described above. Your data will not be processed beyond the purpose of the study.

III Legal basis:
The legal basis for the personal data processed by you as part of the above-mentioned study is your declaration of consent in accordance with Art. 7 and Art. 9 para. 2 lit. a) GDPR and Section 37 para. 1 of the Rhineland-Palatinate State Hospital Act (LKG RLP).

IV. Recipients/categories of recipients:
The data will only be passed on to third parties (persons who are not involved in the above-mentioned study) or published in anonymised form. The results will be published as part of a doctoral thesis. In addition, up to four publications on this study are planned in scientific journals.

V. Storage period and deletion:
- The study-related data will be deleted after the study objective has been achieved, but at the latest after 10 years or in the event of withdrawal of consent, unless longer retention periods are required by law.

VI Rights of data subjects:
You have the following rights regarding your data:

Right to information in accordance with Article 15 GDPR:
You have the right to request information about whether and which of your personal data is processed by us. The right to information may be restricted within the meaning of Section 27 (2) BDSG, i.e. if data is not processed automatically - for example, only in paper form.

Right to rectification in accordance with Articles 16, 19 GDPR:
You have the right to have incorrect personal data concerning you rectified. The right to rectification may be restricted in accordance with Section 27 (2) BDSG.

Right to erasure in accordance with Articles 17, 19 GDPR:
You have the right to request the erasure of personal data concerning you. If you request the deletion of your data, all your personal data will be deleted.

Right to restriction of processing pursuant to Articles 18, 19 GDPR:
You have the right to restrict the processing of personal data concerning you. The right to restrict processing can also be restricted in accordance with Section 27 (2) BDSG.

Right to data portability in accordance with Art. 20 GDPR:
You have the right to receive the personal data concerning you that you have provided to the person responsible for the study. This means that you can request that

Mirjam Gauch (M.Sc.)
Deputy Director of Studies and Speech Therapist
Department of Psychiatry and Psychotherapy
University Hospital Mainz
Untere Zahlbacher Straße 8
55131 Mainz

this data be transmitted either to you or, if technically possible, to another organisation named by you.

Right to object in accordance with Art. 21 GDPR:
You have the right to object to the processing of personal data concerning you. This right may be restricted in accordance with Section 27 (2) BDSG.

VII Automated decision-making
Your personal data will not be subject to decisions based solely on automated processing (e.g. profiling).

VIII. Consequences of non-provision
The provision of personal data is neither legally nor contractually stipulated, nor is it required for the conclusion of a contract. The provision of data does not constitute an obligation. Failure to provide the data has no consequences.

IX. Person responsible
We are responsible for the processing of your data:

Hospital of Johannes Gutenberg University Mainz
represented by the Executive Board
Langenbeckstraße 1, 55131 Mainz
Phone: 06131 17-0
Website: https://www.unimedizin-mainz.de/

You have the right to lodge a complaint with a supervisory authority of your choice. The competent supervisory authority for the University Hospital Mainz is the Rhineland-Palatinate State Commissioner for Data Protection and Freedom of Information:

P.O. Box 30 40, 55020 Mainz
Hintere Bleiche 34, 55116 Mainz
Phone: +49 (0) 6131 208-2449
Fax: +49 (0) 6131 208-2497
Email: poststelle@datenschutz.rlp.de
https://www.datenschutz.rlp.de

Contact details of the Data Protection Officer of the University Hospital Mainz:

Langenbeckstraße 1
55131 Mainz
Tel.: 06131/17-0
Email: datenschutz@unimedizin-mainz.de

Contact information

If you have any questions or wish to assert your rights as a data subject, please do not hesitate to contact me:

Mirjam Gauch

Mirjam Gauch (M.Sc.)
Deputy Director of Studies and Speech Therapist
Department of Psychiatry and Psychotherapy
University Hospital Mainz
Untere Zahlbacher Straße 8
55131 Mainz

Speech therapist, M.Sc. (Healthcare and Nursing)
Department of Psychiatry and Psychotherapy
University Hospital Mainz
Untere Zahlbacher Straße 8, 55131 Mainz
Phone: 06131/17-2474
E-mail: mirjam.gauch@unimedizin-mainz.de

Yours sincerely,

....
Place, date Name, Signature (Deputy Director of Studies)

JG|U UNIVERSITÄTSmedizin. MAINZ

Mirjam Gauch (M.Sc.)
Deputy Director of Studies and Speech Therapist
Department of Psychiatry and Psychotherapy
University Hospital Mainz
Untere Zahlbacher Straße 8
55131 Mainz

Participant information and declaration of consent for the study:
Biography work for persons with PPA

Full title:
The influence of biography work according to the narraktiv protocol by Corsten et al. (2015) on the quality of life of persons with primary progressive aphasia

I hereby declare,

First name and surname :...,
Please complete in block letters.

Date of birth: ....,

Participant code: ....,
(Pseudonym, will be entered by the study director)

I am willing to participate voluntarily in the above-mentioned study. I was informed in a personal interview by
Mr/Mrs/Ms
....
(Name of the study doctor)

I was informed in detail and clearly about the nature, significance, risks and implications of the study. I had the opportunity to have a counselling interview. All my questions were answered satisfactorily and I can ask new questions at any time. I have also read and understood the text of the information leaflet.
I had enough time to make up my mind.
I have received, read and understood a copy of the information sheet and the declaration of consent. The original of the signed declaration of consent will remain at the test centre.
I declare that I am voluntarily willing to participate in the scientific study. I have been comprehensively informed about the processing of my personal data in accordance with Art. 13 GDPR.

<u>DATA PROTECTION:</u>

I have understood and expressly agree to this,

1. **that my personal data required for the purpose of the above-mentioned study will be collected by the study doctor and recorded and processed in pseudonymised form, including on electronic data carriers;**
2. **that the study results will be published in an anonymous form that does not allow any conclusions to be drawn about my person;**

Mirjam Gauch (M.Sc.)
Deputy Director of Studies and Speech Therapist
Department of Psychiatry and Psychotherapy
University Hospital Mainz
Untere Zahlbacher Straße 8
55131 Mainz

3. **that my data will only be processed for the above-mentioned purposes and only by study staff or the named recipients or categories of recipients;**
4. **that video recordings of the individual and group therapies are made in which I can be recognised and which serve to evaluate the biographic-narrative approach and quality assurance. The video recordings are deleted immediately after evaluation.**

I can revoke my consent to participate in the study at any time and without giving reasons (verbally, in writing or in text form) and object to the further processing of my data and samples without incurring any disadvantages. In this case, my personal data will be deleted. My cancellation will only take effect from the date of receipt of the declaration by the Mainz University Medical Centre. It has no retroactive effect, i.e. the processing of my data up to this point in time remains lawful.

- **I would like to be informed about the results following the examination.**
- **I would like the following co-treating person (doctor / speech therapist) to be informed of the results of the examination:**
  ....

....
Place, Date Signature of the study doctor

....
Place, Date Signature of the study participant

if applicable:
....
Place, Date Signature of the legal representative

I hereby declare,

Name of the study doctor ....

of the study participant ....

to have been informed verbally and in writing about the nature, significance and risks of the above-mentioned study, to have answered all questions and to have been given a copy of the study information and the informed consent form.

....
Place, Date Signature of the study doctor

Mirjam Gauch (M.Sc.)
Deputy Director of Studies and Speech Therapist
Department of Psychiatry and Psychotherapy
University Hospital Mainz
Untere Zahlbacher Straße 8
55131 Mainz

Participant information and declaration of consent for the study:
Biography work for persons with PPA

Full title:
The influence of biography work according to the narraktiv protocol by Corsten et al. (2015) on the quality of life of persons with primary progressive aphasia

I hereby declare,

First name and surname :..,
Please complete in block letters.

as a relative of

First name and surname :..,
Please complete in block letters.

Date of birth :..,

Participant code: ....
(Pseudonym, will be entered by the study director)

I agreed to take part in an interview following the above-mentioned study and to report on my experiences from the relatives' perspective.

I was informed by Mr/Mrs ....
(Name of the study doctor)

I was informed in detail and clearly about the nature, significance, risks and implications of the study. I had the opportunity to have a counselling interview. All my questions were answered satisfactorily and I can ask new questions at any time. I have also read and understood the text of the information leaflet.
I had enough time to make up my mind.
I have received, read and understood a copy of the information sheet and the declaration of consent. The original of the signed declaration of consent will remain at the test centre.
I declare that I am voluntarily willing to participate in the scientific study. I have been comprehensively informed about the processing of my personal data in accordance with Art. 13 GDPR.

DATA PROTECTION:

I have understood and expressly agree to this,

Mirjam Gauch (M.Sc.)
Deputy Director of Studies and Speech Therapist
Department of Psychiatry and Psychotherapy
University Hospital Mainz
Untere Zahlbacher Straße 8
55131 Mainz

5. **that my personal data required for the purpose of the above-mentioned study will be collected by the study doctor and recorded and processed in pseudonymised form, including on electronic data carriers;**
6. **that the study results will be published in an anonymous form that does not allow any conclusions to be drawn about my person;**
7. **that my data will only be processed for the above-mentioned purposes and only by study staff or the named recipients or categories of recipients;**
8. **that audio recordings of the interview will be made on which my voice can be heard and which will be used to evaluate the biographic-narrative approach and for quality assurance. The audio recording will be deleted immediately after evaluation.**

I can revoke my consent to participate in the study at any time and without giving reasons (verbally, in writing or in text form) and object to the further processing of my data and samples without incurring any disadvantages. In this case, my personal data will be deleted. My cancellation will only take effect from the date of receipt of the declaration by the Mainz University Medical Centre. It has no retroactive effect, i.e. the processing of my data up to this point in time remains lawful.

- ○ **I would like to be informed about the results following the examination.**

....
Place, Date Signature of the study doctor

....
Place, Date Signature of the care partner

I hereby declare,

Name of the study doctor ....

of the study participant ....

to have been informed verbally and in writing about the nature, significance and risks of the above-mentioned study, to have answered all questions and to have been given a copy of the study information and the informed consent form.

....
Place, Date Signature of the study doctor
